# Exploring the Link Between Social and Economic Instability and COPD: A Cross-Sectional Analysis of the 2022 BRFSS

**DOI:** 10.3390/ijerph22081207

**Published:** 2025-07-31

**Authors:** Michael Stellefson, Min-Qi Wang, Yuhui Yao, Olivia Campbell, Rakshan Sivalingam

**Affiliations:** 1 Department of Health Science, The University of Alabama, Tuscaloosa, AL 35487, USA; okcampbell@crimson.ua.edu (O.C.); rmsivalingam@crimson.ua.edu (R.S.); 2 School of Public Health, University of Maryland, College Park, MD 20742, USA; mqw@umd.edu; 3 Department of Community Medicine and Population Health, The University of Alabama, Tuscaloosa, AL 35487, USA; yyao17@ua.edu

**Keywords:** COPD, social determinants of health, health-related social needs

## Abstract

Despite growing recognition of the role that social determinants of health (SDOHs) and health-related social needs (HRSNs) play in chronic disease, limited research has examined their associations with Chronic Obstructive Pulmonary Disease (COPD) in population-based studies. This cross-sectional study analyzed 2022 Behavioral Risk Factor Surveillance System (BRFSS) data from 37 U.S. states and territories to determine how financial hardship, food insecurity, employment loss, healthcare access barriers, and psychosocial stressors influence the prevalence of COPD. Weighted logistic regression models were used to assess the associations between COPD and specific SDOHs and HRSNs. Several individual SDOH and HRSN factors were significantly associated with COPD prevalence, with financial strain emerging as a particularly strong predictor. In models examining specific SDOH factors, economic hardships like inability to afford medical care were strongly linked to higher COPD odds. Psychosocial HRSN risks, such as experiencing mental stress, also showed moderate associations with increased COPD prevalence. These findings suggest that addressing both structural and individual-level social risks may be critical for reducing the prevalence of COPD in populations experiencing financial challenges.

## 1. Introduction

Chronic Obstructive Pulmonary Disease (COPD) remains a significant global contributor to illness and death, disproportionately affecting individuals with limited access to healthcare and financial resources. Social determinants of health (SDOHs) encompass the broader conditions in which individuals are born, grow, live, and work that influence numerous aspects of health, daily functioning, overall quality of life, and associated risks [1], while health-related social needs (HRSNs) refer to specific social challenges that directly impact individual health outcomes [2]. These factors are shaped by structural inequities and economic policies that systematically disadvantage marginalized populations [3]. Increasing evidence suggests that financial insecurity, employment instability, housing precarity, lack of reliable transportation, and difficulties affording healthcare and basic utilities exacerbate the burden of COPD [4,5,6,7]. Given the interplay between these social risks and health outcomes, understanding how SDOH and HRSN contribute to the prevalence of COPD is critical for informing targeted public health interventions.

COPD disproportionately affects individuals residing in socioeconomically disadvantaged communities, where high disease prevalence often coincides with increased social vulnerability. Research indicates that over 4.5 million adults with COPD live in regions characterized by high levels of economic disadvantage, reinforcing the connection between social environment and respiratory health outcomes [8]. The association between socioeconomic status and COPD is well-documented, with individuals facing financial hardship experiencing limited access to early diagnosis, treatment, and disease management resources [9]. Social support has been identified as a critical determinant of health outcomes in COPD patients, with lower levels of social connectedness correlating with poorer disease management and increased risk of hospitalization [6,10]. Furthermore, individuals experiencing food insecurity and emotional distress face an increased likelihood of experiencing adverse health effects, suggesting that the intersection of SDOH and HRSN plays a vital role in COPD disparities [11,12].

Economic instability further exacerbates COPD risk, as financial hardship limits healthcare access and contributes to heightened stress and poor disease management [13]. Many adults with COPD face significant economic challenges, including lower household incomes [14], early retirement due to disability [15], and higher rates of unemployment [14]. Financial strain is a common concern, with individuals with COPD being significantly more likely to experience economic insecurity by the end of the month and struggle to afford necessary medications [15,16]. Additionally, employment limitations due to COPD-related physical impairments result in reduced productivity and increased absenteeism, further compounding economic burdens [14,15]. These financial stressors not only undermine quality of life but may also lead to a vicious cycle of worsening health outcomes, as individuals with limited financial resources may forgo necessary medical care and treatment adherence [17].

Limited access to nutritious food and reliable transportation has also been identified as a key factor contributing to COPD-related health disparities. Many adults with COPD experience food insecurity [16], which restricts their ability to access healthy foods necessary for managing their condition [7]. Food insecurity is closely tied to poor dietary quality, which can exacerbate respiratory symptoms and contribute to increased COPD exacerbations [18]. Compounding this issue, many individuals with COPD report significant transportation challenges, including mobility limitations, financial barriers to travel, and inadequate public transportation options [19]. These barriers not only affect access to nutritious food but also limit the ability to attend medical appointments and engage in preventive care. Consequently, individuals with COPD who face food and transportation insecurity may experience worse disease management outcomes and increased hospitalization rates [20].

Despite the growing recognition of the role of SDOH and HRSN in shaping COPD prevalence, only limited research has examined these associations using population-based data. Hacker et al. [21] utilized data from the Behavioral Risk Factor Surveillance System’s (BRFSS) Social Determinants and Health Equity module, which encompassed responses from 39 states, the District of Columbia, and two US territories, to investigate adults with chronic respiratory diseases, including COPD. Individuals with COPD experienced notably higher rates of food insecurity, utility shutoff threats, and social isolation compared to those without the disease, illustrating the broad impact of social disadvantage on respiratory health. People with COPD were also significantly more likely to report having two to five or more adverse SDOH and HRSN compared to adults without this condition. Cost barriers to medical care emerged as the strongest individual predictor of COPD, followed by mental stress and lack of reliable transportation.

The current study aimed to build on the work of Hacker et al. [21] by focusing exclusively on COPD. Unlike this previous research, which modeled all SDOH and HRSN variables simultaneously and reported prevalence ratios for a broad set of chronic diseases, this study employed a series of stratified logistic regression models to estimate adjusted odds ratios for COPD specifically. By independently evaluating the effects of SDOH and HRSN risk factors in separate models, this study introduced a more nuanced assessment of how distinct dimensions of social disadvantage contribute to COPD prevalence.

## 2. Materials and Methods

### 2.1. Research Design

The Behavioral Risk Factor Surveillance System (BRFSS) is a large-scale public health survey conducted by the Centers for Disease Control and Prevention (CDC) that plays a vital role in gathering information on chronic conditions, health risk behaviors, and the use of preventive services across the US. In 2022, the BRFSS expanded its scope to include a detailed assessment of SDOH and HRSN, which are included in an optional module (Module 16) that explores these determinants and health equity.

The cross-sectional study sample represents a broad demographic range. These states and territories include Alabama, Alaska, Arizona, California, Connecticut, Delaware, District of Columbia, Florida, Georgia, Idaho, Indiana, Iowa, Kansas, Kentucky, Maine, Massachusetts, Minnesota, Mississippi, Missouri, Montana, Nevada, New Hampshire, New Jersey, New Mexico, North Carolina, Puerto Rico, Rhode Island, South Carolina, Tennessee, Texas, Utah, Vermont, Virgin Islands, Washington, West Virginia, Wisconsin, and Wyoming. The BRFSS uses a stratified sampling approach to ensure that the data accurately represents the adult population of each participating state [22]. The respondents are interviewed by phone in both English and Spanish, making the survey more accessible and inclusive [23]. The BRFSS survey gathers self-reported data that helps researchers understand the experiences of individuals with various chronic diseases, particularly in relation to their social environments and health-related needs [21,24].

### 2.2. Measures

Respondents who completed the optional SDOH module of the BRFSS provided data on a range of demographic, socioeconomic, and health-related measures. Demographic information included gender (male and female), age group (18–34, 35–44, 45–54, 55–64, 65–74, and 75+), race/ethnicity (White, Black, Other race, Multiracial, and Hispanic), veteran status (yes or no), and the resident states mentioned previously. Socioeconomic factors were assessed through marital status (married, previously married, and never married), household income (USD 0 to USD 24,999, USD 25,000 to USD 34,999, USD 35,000 to USD 49,999, USD 50,000 to USD 99,999, and USD 100,000+), education level (less than high school, high school graduate, some college, and college graduate), employment status (employed, unemployed, and retired), and homeownership (own or do not own).

In addition to asking respondents whether or not they had COPD, respondents also reported if they had other health conditions, including heart disease or myocardial infarction (MI), asthma, depressive disorder, diabetes, arthritis, and multimorbidity (the presence of multiple chronic conditions). Also, other health behaviors, including smoking, obesity, heavy alcohol use, and physical activity, are included in the analyses. Missing responses (“Do not know” or “Refused”) were treated as missing data and excluded from the analyses.

The SDOHs and HRSNs included in this study were assessed using validated items from the Behavioral Risk Factor Surveillance System’s (BRFSS) Social Determinants and Health Equity module [25]. These items were initially derived from an HRSN screening tool created by the Centers for Medicare & Medicaid Services’ Innovation Center [26]. Two additional SDOH indicators, health insurance coverage and cost as a barrier to needed medical care, were derived from the BRFSS Health Care Access Core Section. Each SDOH indicator was operationalized dichotomously (1 = presence of adverse condition; 0 = absence of adverse condition). The specific SDOH assessed included employment instability, defined as losing a job or experiencing reduced work hours in the past 12 months; housing insecurity, indicated by the inability to pay rent, mortgage, or utility bills; and food insecurity, measured by respondents reporting that the food they bought did not last and they did not have money to purchase more. Other SDOH indicators included experiencing a threat of utility shutoff; having no health insurance coverage; lacking reliable transportation to access work, services, or medical appointments; and being unable to afford a doctor’s visit when needed.

HRSNs were measured using questions that captured psychosocial challenges and access to community-based supports [27]. Life dissatisfaction was identified when respondents reported being “dissatisfied” or “very dissatisfied” with their lives. Lack of social and emotional support was defined as receiving such support only “sometimes,” “rarely,” or “never.” Social isolation was measured by the frequency of feeling socially isolated, with responses of “always,” “usually,” or “sometimes” indicating risk. Mental stress was assessed using a single-item measure that asked how often respondents experienced significant stress, defined as feeling tense, restless, nervous, or unable to sleep due to constant worry. Responses of “always” or “usually” were classified as high stress. Additionally, the use of food assistance was included as an indicator, with respondents reporting receipt of Supplemental Nutrition Assistance Program (SNAP) benefits within the past 12 months coded as having a relevant HRSN.

### 2.3. Data Analysis

Data analysis procedures were designed and conducted to comprehensively assess the associations between COPD, SDOH, and HRSN, accounting for the BRFSS’s complex sampling design and applying survey weights to yield findings for the broader US population. Demographic, socioeconomic, health condition, and health behavior covariates were presented using both unweighted and weighted samples, each with corresponding 95% confidence intervals (CIs). Rao–Scott chi-square tests were performed to assess the association between COPD and each covariate: demographic characteristics (gender, age group, race/ethnicity, veteran status, and marital status), socioeconomic position indicators (education level, household income, current employment status, and homeownership), co-occurring health conditions (heart disease, asthma, depressive disorder, diabetes, arthritis, and multimorbidity) and health behavior (smoking, obesity, heavy alcohol use, and physical activity). Three multivariable weighted logistic regression models were conducted to estimate the independent associations of COPD prevalence with (1) SDOH risk factors, (2) HRSN risk factors, and (3) both SDOH and HRSN risk factors combined. The models were adjusted for the covariates and yielded adjusted odds ratios (AORs) with 95% CIs to quantify their association with COPD prevalence. The analyses were performed in SAS Version 9.4, utilizing its survey-specific procedures [28,29] to handle complex survey data appropriately. Although the resident state was included as a demographic covariate in all analyses, it was omitted from the display to conserve space.

## 3. Results

Table 1 reveals the characteristics of the sample after applying survey weights. The weighted sample was nearly evenly split between males (49.27%) and females (50.73%), with a notable proportion of respondents aged 18–34 years (28.08%). The majority identified as White and non-Hispanic (59.87%), while Hispanic respondents represented 17.78%. Educational attainment was high, with 32.27% being college graduates. The income distribution showed that 28.98% of the population earned USD 100,000 or more annually. Most respondents were employed (66.61%), and 69.42% reported owning a home. Health conditions varied, with 27.14% diagnosed with arthritis, 21.57% reporting depressive disorders, 15.47% having asthma, and 8.97% reporting heart disease or myocardial infarction. Notably, more than one out of five (21.44%) respondents reported multimorbidity (two or more chronic conditions). Health behaviors show that most respondents engaged in no physical activity (76.08%), yet they were non-smokers (87.81%), abstained from heavy alcohol use (93.39%), and were not obese (67.47%).

Table 2 presents the results of the Rao–Scott chi-square test examining the associations between self-reported COPD diagnoses and demographic, socioeconomic, and health condition covariates. The COPD prevalence differed significantly across all variables (all *p* < 0.0001). Several noteworthy findings were revealed when COPD was presented. Females (4.54%) had a higher COPD prevalence than males (3.34%). Non-Hispanic White individuals reported the highest COPD prevalence (6.39%) compared to other racial/ethnic groups. Socioeconomic disparities were also evident, with higher COPD rates among those earning less than USD 25,000 annually and among individuals with less than a high school education. The COPD prevalence was markedly higher among individuals with co-occurring heart disease or myocardial infarction (5.75% vs. 2.04%), asthma (4.74% vs. 3.14%), depressive disorders (4.81% vs. 3.07%), and diabetes (5.83% vs. 2.05%). Among the respondents with COPD, the most frequently reported health behaviors were not smoking (4.59% vs. 2.28%), abstinence from heavy drinking (6.36% vs. 0.47%), physical inactivity (3.84% vs. 2.92%), and obesity (4.92% vs. 2.01%).

Table 3 presents AORs estimating the association between individual SDOH risk factors and the self-reports of a COPD diagnosis, adjusting for relevant demographic, socioeconomic, health condition, and health behavior covariates. Among the seven SDOH indicators included, one was significantly associated with higher odds of COPD. Individuals who reported being unable to afford medical costs had greater odds of reporting COPD (AOR = 1.49; 95% CI: 1.16, 1.90). Other SDOH indicators, including job loss or reduced work hours, food insecurity, inability to pay bills, utility shutoffs, lack of reliable transportation, and lack of health insurance, were not significantly associated with COPD after adjustment for these factors.

Table 4 presents AORs assessing the association between individual HRSN and the self-reports of a COPD diagnosis after adjusting for covariates. Individuals who reported experiencing frequent stress appeared to have higher odds of having COPD (AOR = 1.19; 95% CI: 1.01, 1.40). In contrast, other HRSN indicators, such as dissatisfaction with life, lack of emotional support, social isolation, and being on food stamps, were not significantly associated with COPD prevalence in the adjusted model.

Table 5 presents AORs estimating the association between COPD prevalence and both SDOH and HRSN when modeled simultaneously. Among the individual SDOH and HRSN indicators, only the inability to afford medical costs (could not afford medical costs) was significantly associated with higher odds of COPD (AOR = 1.49; 95% CI: 1.15, 1.93).

## 4. Discussion

Our study found that both individual measures of social disadvantage were significantly associated with COPD prevalence. Key demographic disparities emerged, with higher COPD rates among women, older adults, non-Hispanic White individuals, and those with lower income and education levels. Several individual SDOH and HRSN factors, particularly financial strain and stress, were linked to increased odds of COPD. These results illustrate the complex, multifactorial nature of COPD risk and highlight the importance of addressing both economic and psychosocial dimensions of health in managing chronic respiratory disease.

When examining the subgroup of individuals with COPD, the findings reveal significant differences in demographic factors. Females had a higher prevalence of COPD (4.54%) compared to males (3.34%), aligning with previous studies that highlight a higher burden of COPD among women [30]. Women were found to have a higher burden of COPD than men, which aligns with previous research indicating that women experience more severe disease progression despite lower cumulative smoking exposure [31,32]. Women with COPD typically experience more extended hospital stays, increased rates of hospital readmission, and a higher occurrence of comorbidities compared to men. Yet, they may be undertreated despite their higher disease burden and hospitalization rates [31].

Additionally, the prevalence of COPD increased notably with age, peaking among those aged 65–74, supporting the existing literature that links age to a higher risk of chronic respiratory diseases [33]. Racial and ethnic disparities were also evident, as non-Hispanic White individuals reported the highest COPD prevalence (6.39%), consistent with studies showing higher rates of chronic respiratory diseases in this group [34]. Moreover, socioeconomic disparities were apparent, with individuals from lower-income backgrounds and those with lower educational attainment experiencing higher rates of COPD, reinforcing the role of SDOH in the exacerbation of chronic conditions [35,36,37].

In the disaggregated model SDOH, inability to afford medical care was significantly associated with higher odds of COPD, indicating that economic inequality remains a significant barrier to respiratory health. This aligns with previous research linking financial stress to adverse health outcomes, including chronic respiratory diseases [5,38,39,40,41]. Individuals under financial strain may prioritize immediate survival needs over long-term disease management, which can lead to poorer health outcomes [42]. Future analyses should explore potential interaction effects between SDOH and key demographic characteristics (e.g., age) to yield a more nuanced understanding of how these factors differentially influence COPD prevalence across population subgroups.

Among HRSN, stress was significantly associated with COPD, although its *p*-value did not meet the Bonferroni-adjusted significance threshold. These findings underscore the impact of psychosocial stress in shaping disease burden [43]. The significant association underscores the impact of psychological stress on the management and progression of chronic illness [44,45]. Interventions such as peer support, counseling, and community-based programs may help alleviate the psychological toll experienced by individuals living with COPD [46].

When SDOH and HRSN variables were included together in a combined model (Table 5), only the inability to afford medical care remained statistically significant, suggesting that it may be uniquely linked to COPD after accounting for a wider array of social disadvantages [21]. This result supports existing evidence that economic hardship plays a critical role in the onset and progression of chronic diseases such as COPD [11,47].

Notably, a lack of reliable transportation was identified by Hacker et al. [21] as an important predictor of COPD in their combined HRSN/SDOH model; however, it was not significant in the current study. Several methodological differences may explain this discrepancy. First, the current study included a smaller number of US states and territories, which may have reduced the generalizability and statistical power needed to detect associations for certain risk factors. Second, differences in covariate adjustment may have influenced the results. The current study adjusted for a broad set of chronic health conditions (i.e., heart attack/coronary heart disease, asthma, depressive disorder, diabetes, arthritis, multimorbidity) and disease-specific behavioral risk factors [48]. Additionally, by separately analyzing the impacts of SDOHs and HRSNs in individual models, this study provided a more refined understanding of how different aspects of social disadvantage are linked to the prevalence of COPD. These differences in the analytic approach may have impacted the observed associations and emphasize the importance of model specification when interpreting the influence of social risks on COPD.

### Limitations

As this was a cross-sectional study, there is a possibility that the direction of associations is reversed. For example, individuals with COPD may be more likely to experience adverse SDOH or HRSN. Also, the use of self-reported COPD diagnosis from the BRFSS survey, based on the question “Have you been diagnosed by a physician as having COPD or emphysema?”, may underestimate the true COPD prevalence. Although this type of question has demonstrated high specificity and positive predictive value, its low sensitivity, particularly for detecting mild or undiagnosed cases [49], suggests that individuals with less severe disease may go unrecognized. As a result, this study may disproportionately reflect adults with more advanced symptoms or greater engagement with the healthcare system.

In addition, the BRFSS survey’s reliance on random-digit dialing introduces potential selection bias, as individuals who screen calls, rely exclusively on mobile phones, or lack phone access may be underrepresented. This selection bias could skew the sample toward individuals who are more reachable via traditional telephone methods and potentially more socially or economically stable. Additionally, because data were only available from a subset of US states and territories that administered the optional SDOH and HRSN modules, the findings may not be generalizable. Our focus on the U.S. population using BRFSS data restricts the generalizability of findings to lower- and middle-income countries, where household air pollution and different smoking prevalence patterns contribute substantially to COPD risk. Finally, because the study window is limited to 2022, the data from other years are excluded, potentially introducing bias.

Although smoking was incorporated into all multivariable models as a dichotomous variable (current versus non-smoker) to address confounding, our reliance on self-reported smoking data may introduce misclassification bias and limit the ability to capture lifetime exposure or intensity of smoking behaviors. Additionally, while we included unweighted counts alongside weighted percentages to improve transparency, residual confounding from unmeasured factors (e.g., environmental exposures or occupational hazards) may persist.

## 5. Conclusions

Individual social risk factors were significantly associated with COPD prevalence, with financial strain emerging as a key contributor. In disaggregated models, the inability to afford medical costs was significantly linked to increased COPD odds, underscoring the role of economic hardship as a significant barrier to respiratory health. Among HRSN variables, stress was also associated with higher COPD prevalence, highlighting the impact of psychosocial strain and potential reliance on social support. However, when all SDOH and HRSN factors were modeled simultaneously, only the inability to afford medical care remained significant, pointing to its unique and robust linkage to COPD risk. These results support the importance of implementing broad public health initiatives that address both economic and psychosocial dimensions of social disadvantage in COPD. Future studies should investigate the effectiveness of integrated care models that address both medical and non-medical determinants of COPD, as well as policy-level interventions aimed at reducing financial barriers to treatment.

## Figures and Tables

**Table 1:** Unweighted and weighted frequencies of demographics, socioeconomic position, health conditions, and health behaviors of the BRFSS respondents who completed the SDOH module, 2022.

Characteristic	Unweighted Sample, *n* (%)	Weighted Sample, *N* (%)	Weighted 95% CI (%)
***DEMOGRAPHICS***			
**Gender**			
Male	157,448 (47.66)	93,591,795 (49.27)	48.91–49.63
Female	172,909 (52.34)	96,364,789 (50.73)	50.37–51.09
**Age Group**			
18–34	54,626 (16.54)	53,344,348 (28.08)	27.74–28.43
35–44	46,982 (14.22)	33,213,110 (17.48)	17.20–17.77
45–54	50,150 (15.18)	29,452,496 (15.50)	15.24–15.77
55–64	63,557 (19.24)	32,152,662 (16.93)	16.66–17.19
65–74	68,202 (20.64)	25,631,966 (13.49)	13.27–13.71
75+	46,840 (14.18)	16,162,003 (8.51)	8.32–8.69
**Race/Ethnicity**			
White Only, Non-Hispanic	248,327 (75.17)	113,730,000 (59.87)	59.49–60.26
Black Only, Non-Hispanic	26,392 (7.99)	22,329,393 (11.75)	11.51–12.00
Other Race Only, Non-Hispanic	16,818 (5.09)	14,153,864 (7.45)	7.21–7.69
Multiracial, Non-Hispanic	7508 (2.27)	5,968,374 (3.14)	3.00–3.28
Hispanic	31,311 (9.48)	33,775,630 (17.78)	17.43–18.13
**Veteran Status**			
Yes	41,235 (12.48)	19,916,158 (10.48)	10.28–10.69
No	289,120 (87.52)	170,040,000 (89.52)	89.31–89.72
**Marital Status**			
Married or Member of Unmarried Couple	188,479 (57.05)	108,250,000 (56.99)	56.63–57.34
Previously Married	84,659 (25.63)	37,584,745 (19.79)	19.52–20.06
Never Married	57,218 (17.32)	44,124,541 (23.23)	22.91–23.55
***SOCIOECONOMIC POSITION***			
**Education**			
Less than HS	16,556 (5.01)	19,220,084 (10.12)	9.83–10.41
HS Graduate	77,716 (23.52)	50,545,213 (26.61)	26.28–26.94
Some College	90,846 (27.50)	58,886,028 (31.00)	30.66–31.34
College Grad	145,239 (43.96)	61,305,260 (32.27)	31.96–32.58
**Income**			
USD 0 to USD 24,999	51,900 (15.71)	31,868,141 (16.78)	16.49–17.06
USD 25,000 to USD 34,999	39,518 (11.96)	23,514,055 (12.38)	12.13–12.62
USD 35,000 to USD 49,999	44,244 (13.39)	24,436,729 (12.86)	12.62–13.11
USD 50,000 to USD 99,999	102,446 (31.01)	55,089,728 (29.00)	28.68–29.32
USD 100,000+	92,249 (27.92)	55,047,931 (28.98)	28.65–29.31
**Employment**			
Employed	180,377 (58.01)	115,470,000 (66.61)	66.26–66.96
Unemployed	31,294 (10.06)	20,595,541 (11.88)	11.63–12.13
Retired	99,278 (31.93)	37,287,446 (21.51)	21.22–21.80
**Home Ownership**			
Own	236,588 (71.62)	131,880,000 (69.42)	69.10–69.75
Do Not Own	93,769 (28.38)	58,079,519 (30.58)	30.25–30.90
***HEALTH CONDITIONS***			
**Heart Disease or MI**			
Yes	29,384 (8.97)	12,988,458 (6.89)	6.72–7.06
No	298,342 (91.03)	175,460,000 (93.11)	92.94–93.28
**Asthma**			
Yes	50,437 (15.27)	29,387,376 (15.47)	15.21–15.73
No	279,920 (84.73)	160,570,000 (84.53)	84.27–84.79
**Depressive Disorder**			
Yes	71,465 (21.63)	40,976,100 (21.57)	21.28–21.86
No	258,890 (78.37)	148,980,000 (78.43)	78.14–78.72
**Diabetes**			
Yes	45,614 (13.81)	23,010,437 (12.11)	11.88–12.35
No	284,743 (86.19)	166,950,000 (87.89)	87.65–88.12
**Arthritis**			
Yes	112,914 (34.31)	51,326,882 (27.14)	26.83–27.45
No	216,178 (65.69)	137,810,000 (72.86)	72.55–73.17
**Multimorbidity**			
Yes	83,115 (25.16)	40,725,720 (21.44)	21.16–21.72
No	247,242 (74.84)	149,230,000 (78.56)	78.28–78.84
***HEALTH BEHAVIORS***			
**Smoking**			
Yes	49,941 (12.19)	30,780,191 (12.84)	12.62–13.05
No	359,729 (87.81)	209,006,948 (87.16)	86.95–87.38
**Heavy Alcohol Use**			
Yes	26,305 (6.65)	15,166,528 (6.61)	6.45–6.77
No	369,122 (93.35)	214,359,223 (93.39)	93.23–93.55
**Physical Activity**			
Yes	106,480 (23.98)	63,161,067 (23.92)	23.65–24.20
No	337,559 (76.02)	200,855,153 (76.08)	75.80–76.35
**Obesity**			
Yes	272,572 (68.77)	155,255,414 (67.47)	67.15–67.78
No	123,754 (31.23)	74,863,547 (32.53)	32.22–32.85

**Table 2:** Self-report of a healthcare professional diagnosis of COPD by socio-demographic factors and comorbid health conditions, BRFSS 2022.

Characteristic	% With COPD	% Without COPD	*p* Value (Rao–Scott Chi Square)
	7.99	92.01	<0.0001
***DEMOGRAPHICS***			
**Gender**			<0.0001
Male	3.34	44.06	
Female	4.54	48.06	
**Age Group**			<0.0001
18–34	0.32	15.98	
35–44	0.46	13.94	
45–54	0.85	14.51	
55–64	1.90	17.38	
65–74	2.50	18.25	
75+	1.86	12.06	
**Race/Ethnicity**			<0.0001
White Only, Non-Hispanic	6.39	69.12	
Black Only, Non-Hispanic	0.61	7.27	
Other Race Only, Non-Hispanic	0.26	4.80	
Multiracial, Non-Hispanic	0.24	2.02	
Hispanic	0.39	8.91	
**Veteran Status**			<0.0001
Yes	1.43	11.03	
No	6.45	81.09	
**Marital Status**			<0.0001
Married or Member of Unmarried Couple	3.40	54.29	
Previously Married	3.56	21.76	
Never Married	0.92	16.07	
***SOCIOECONOMIC POSITION***			
**Education**			<0.0001
Less than HS	0.798	3.95	
HS Graduate	2.54	20.57	
Some College	2.64	24.78	
College Grad	1.92	42.81	
**Income**			<0.0001
USD 0 to USD 24,999	2.67	12.43	
USD 25,000 to USD 34,999	1.41	10.36	
USD 35,000 to USD 49,999	1.18	12.12	
USD 50,000 to USD 99,999	1.84	29.46	
USD 100,000+	0.79	27.75	
**Employment**			<0.0001
Employed	2.12	56.45	
Unemployed	2.04	7.67	
Retired	3.92	27.80	
**Home Ownership**			<0.0001
Own	5.29	66.83	
Do Not Own	2.59	25.28	
***HEALTH CONDITIONS***			
**Heart Disease or MI**			<0.0001
Yes	5.75	85.41	
No	2.04	6.80	
**Asthma**			<0.0001
Yes	4.74	80.07	
No	3.14	12.05	
**Depressive Disorder**			<0.0001
Yes	4.81	73.61	
No	3.07	18.51	
**Diabetes**			<0.0001
Yes	5.83	80.46	
No	2.05	11.66	
**Arthritis**			<0.0001
Yes	2.84	62.96	
No	5.03	29.17	
**Multimorbidity**			<0.0001
Yes	3.01	72.02	
No	4.87	20.10	
***HEALTH BEHAVIORS***			
**Smoking**			<0.0001
Yes	2.28	10.51	
No	4.59	82.62	
**Heavy Alcohol Use**			0.2550
Yes	0.47	6.14	
No	6.36	87.03	
**Physical Activity**			<0.0001
Yes	2.92	20.95	
No	3.84	72.29	
**Obesity**			<0.0001
Yes	4.92	62.55	
No	2.01	30.51	

**Table 3:** Adjusted odds ratios for COPD prevalence by SDOH risks.

SDOH Risk	Having COPD/OR (95% CI)
Lost employment or had hours reduced	1.16 (0.86, 1.55)
Food insecurity	1.20 (0.94, 1.54)
Inability to pay bills	1.05 (0.81, 1.38)
Not able to pay utility bills	1.13 (0.84, 1.51)
Lack of reliable transportation	0.90 (0.69, 1.17)
No health insurance	0.71 (0.49, 1.03)
Inability to afford medical costs	1.49 ** (1.16, 1.90)

Notes: Data are presented as weighted odds ratios (95% CI), adjusted for demographic characteristics (gender, age group, race/ethnicity, veteran status, marital status, and resident state), socioeconomic position (educational attainment, income, current employment status, and homeownership), health condition (heart attack/coronary heart disease, asthma, depressive disorder, diabetes, arthritis, and multimorbidity), and health behavior (smoking, obesity, heavy alcohol use, and physical activity). The overall test’s *p*-values, based on the likelihood ratio, score, and Wald tests, were all <0.0001. ** *p* ≤ 0.0071, where 0.0071 is the Bonferroni-adjusted significance levels.

**Table 4:** Adjusted odds ratios for COPD prevalence by HRSN risks.

HRSN Risk	Having COPD/OR (95% CI)
Dissatisfaction with life	0.97 (0.75, 1.24)
Lack of Emotional support	0.97 (0.74, 1.27)
Social Isolation	1.06 (0.89, 1.26)
Stress	1.19 * (1.01, 1.40)
Food Stamps	1.18 (0.93, 1.51)

Notes: Data are presented as weighted odds ratios (95% CI), adjusted for demographic characteristics (gender, age group, race/ethnicity, veteran status, marital status, and resident state), socioeconomic position (educational attainment, income, current employment status, and homeownership), health condition (heart attack/coronary heart disease, asthma, depressive disorder, diabetes, arthritis, and multimorbidity), and health behavior (smoking, obesity, heavy alcohol use, and physical activity). The overall test’s *p*-values, based on the likelihood ratio, score, and Wald tests, were all <0.0001. * *p* ≤ 0.05.

**Table 5:** Adjusted odds ratios for COPD prevalence by SDOH and HRSN risks.

SDOH and HRSN Risk Factors	Having COPD/OR (95% CI)
***SDOH Risk***	
Lost employment or had hours reduced	1.13 (0.84, 1.52)
Food insecurity—no money for food when the food went bad	1.20 (0.93, 1.56)
Inability to pay bills	1.02 (0.77, 1.34)
Not able to pay utility bills	1.14 (0.84, 1.54)
Lack of reliable transportation	0.89 (0.68, 1.17)
No health insurance	0.72 (0.49, 1.05)
Inability to afford medical costs	1.49 (1.15, 1.93) **
***HRSN Risk***	
Dissatisfaction with life	0.94 (0.74, 1.20)
Lack of Emotional support	0.92 (0.69, 1.22)
Social Isolation	1.08 (0.91, 1.29)
Stress	1.11 (0.93, 1.32)
Food Stamps	1.17 (0.90, 1.52)

Notes: Data are presented as weighted odds ratios (95% CI), adjusted for demographic characteristics (gender, age group, race/ethnicity, veteran status, marital status, and resident state), socioeconomic position (educational attainment, income, current employment status, and homeownership), health condition (heart attack/coronary heart disease, asthma, depressive disorder, diabetes, arthritis, and multimorbidity), and health behavior (smoking, obesity, heavy alcohol use, and physical activity). The overall test’s *p*-values, based on the likelihood ratio, score, and Wald tests, were all <0.0001. ** *p* ≤ 0.0042, where 0.0042 is the Bonferroni-adjusted significance levels.

## Data Availability

Publicly available datasets were analyzed in this study. The BRFSS data analyzed during this study can be found at the following: https://www.cdc.gov/brfss/annual_data/annual_2022.html (accessed on 10 April 2025).
